# Underinvesting in prevention: the costly blind spot in US diabetes care

**DOI:** 10.1093/haschl/qxag190

**Published:** 2026-07-18

**Authors:** Paul Y Han

**Affiliations:** Department of Diabetes, Endocrinology & Metabolism, City of Hope National Medical Center, Duarte, CA 91010, United States; Department of Surgery, South Coast Global Medical Center, Santa Ana, CA 92704, United States

**Keywords:** diabetes, Podiatry, diabetic foot, amputation prevention, preventive care, health policy, value-based care, multidisciplinary care, diabetes complications, health care costs

## Abstract

To address the systemic financial and equity burdens of preventable diabetes-related lower-extremity amputations, this perspective evaluates current structural failures in US healthcare financing and proposes a scalable, prevention-oriented framework. Current fee-for-service models consistently reimburse expensive disease deterioration while neglecting early surveillance. Meanwhile, omitting preventive foot metrics from federal quality frameworks renders early intervention organizationally invisible. In response, this analysis incorporates data from a peer-reviewed, 5-year implementation study within a medically complex population using low-overhead, multilingual, digital patient education and risk-stratified surveillance. This low-friction intervention model achieved a diabetic foot ulcer rate of 2.8% and a major amputation rate of 0.43%, significantly outperforming the federal Healthy People 2030 benchmark of 0.55%. To replicate these results nationally, public payers must integrate risk-stratified podiatric surveillance into core quality reporting frameworks, expand reimbursement codes for asynchronous digital education, and formally classify patient-centered education as vital health infrastructure. Such policy prioritization will mitigate profound health disparities, reduce avoidable public spending, and preserve patient mobility.

## Introduction

A blister, painless callus, change in skin texture, or subtle painless change in the shape of the foot and appearance may seem clinically insignificant in the early stages of diabetes-related foot disease. Yet delayed recognition of these warning signs frequently initiates a cascade of events culminating in infection, hospitalization, lower-extremity amputation, disability, and premature mortality.^1,2^ Diabetic foot complications remain among the most preventable yet costly manifestations of diabetes, generating substantial expenditures across emergency care, inpatient hospitalization, post-acute rehabilitation, long-term care, and disability services and payment.^3^

Despite advances in diabetes management, preventable diabetic limb loss remains persistently common. This persistence raises an important policy question: Why does the US healthcare system continue paying for preventable deterioration rather than systematically investing in prevention?

Resources are often mobilized only after clinical deterioration becomes visible and expensive. In contrast, preventive interventions—including symptom recognition, routine surveillance, timely podiatric assessment, patient education, and coordinated referral pathways—are fragmented, inconsistently incentivized, and frequently inaccessible to high-risk populations.^4^ Preventable diabetic limb loss should be understood not primarily as an isolated clinical complication, but as a systems-level failure in prevention policy, payment design, and care delivery. To demonstrate the viability of systemic reform, this perspective leverages outcomes from a 5-year implementation study^5^ to outline a scalable policy framework for the Centers for Medicare & Medicaid Services (CMS) and state Medicaid agencies.

## Underfunding and structural misalignment

Contemporary diabetes care successfully emphasizes several preventive domains, including glycemic control, retinal screening, nephropathy monitoring, and medication management. However, preventive limb preservation remains comparatively underemphasized in policy design and payment incentives.^1,2^ Current reimbursement structures reward the treatment of complications more predictably than their prevention. Hospital admissions, wound care procedures, vascular interventions, and amputations are highly reimbursable and operationally visible.^3^ In contrast, prevention relies upon fragmented encounters and longitudinal coordination that may not fit neatly into existing reimbursement pathways.

Furthermore, responsibility for preventive surveillance is diffuse and poorly coordinated across primary care physicians, endocrinologists, podiatrists, and home caregivers. This fragmentation is particularly severe within resource-limited safety-net health centers.^4^ For policymakers focused on efficiency and value, this represents a preventable systems failure rather than an unavoidable clinical inevitability.

This structural deficit is further compounded by a mismatch between investment timelines and reimbursement incentives. Preventive investments may require several years before downstream savings become visible. Health plans operating within annual budgeting cycles often struggle to justify prevention expenditures when avoided costs emerge later. However, the policy objective is not merely cost containment but the preservation of mobility, independence, and quality of life.

## A health equity and quality measurement failure

The burden of diabetic limb loss is disproportionately borne by Medicaid beneficiaries, rural populations, residents of long-term care facilities, and racial and ethnic minority communities.^1,2^ Social determinants of health—including transportation barriers, language discordance, and low health literacy—delay preventive intervention and reduce opportunities for early recognition. Safety-net systems frequently care for patients with a high diabetes burden but limited specialty access, a reality reflected in databases such as the Uniform Data System.^4^

This visibility problem is driven directly by quality measurement frameworks. Diabetes quality programs emphasize glycated hemoglobin levels, retinal exams, and blood pressure control. However, preventive foot surveillance remains less systematically incorporated. Health systems prioritize what is measured, reported, and publicly benchmarked. When early foot-risk identification remains invisible within federal performance architectures such as the Merit-based Incentive Payment System (MIPS)^6^ and the Healthcare Effectiveness Data and Information Set (HEDIS),^7^ preventive efforts receive lower organizational attention.^1-3^ What remains unmeasured remains underprioritized.

## Scalable models of preventive care delivery

Encouragingly, scalable preventive approaches already exist and do not require expensive infrastructure or burdensome clinical workflows. The empirical viability of a low-friction approach is illustrated by a 5-year diabetic limb preservation initiative implemented in a medically complex, highly vulnerable population of patients with diabetes undergoing concurrent cancer treatment. By leveraging QR codes at the point of care, the program bypassed traditional technological barriers without disrupting clinical workflows. Instead of generic resources, it provided access to bespoke, multilingual educational materials featuring physicians explaining actual, real-world cases of patients with specific, identifiable conditions. The peer-reviewed findings, published in the *Journal of the American Podiatric Medical Association*,^5^ underscore the macroeconomic and clinical viability of this framework: over the 5-year tracking period, the incidence of diabetic foot ulcers within the program population was approximately 2.8%, compared with commonly reported national rates of 5%-10%.^2,3,5^ Furthermore, major amputation rates dropped to 0.43%—significantly outperforming the federal Healthy People 2030 benchmark of 0.55% established by the US Department of Health and Human Services.^8^

## Policy recommendations

Several systemic policy reforms merit immediate consideration to bridge the gap between treatment and prevention:

- **Incorporate preventive foot metrics into quality programs**: CMS and state Medicaid programs should integrate risk-stratified podiatric surveillance into MIPS,^6^ HEDIS,^7^ and Federally Qualified Health Center (FQHC) core reporting metrics.^1,2^ Elevating early foot-risk identification within performance frameworks will compel health systems to prioritize early intervention.
- **Expand reimbursement for asynchronous and educational surveillance**: Commercial and public payers should establish distinct reimbursement codes for culturally tailored, multilingual digital education pathways used at the point of care to ensure providers can justify upfront investments.^3,5^
- **Classify multilingual complication education as health infrastructure**: Health authorities should formally recognize patient-facing diabetes education as core preventive infrastructure rather than optional programming, particularly in resource-limited safety-net settings where language and health literacy barriers drive disproportionate amputation rates.
- **Fund prevention-first demonstration programs through public payers**: CMS and state Medicaid agencies should launch targeted demonstration programs to evaluate low-burden, prevention-first delivery models that track total cost of care reduction across vulnerable populations using national reporting systems.^4^
- **Elevate limb preservation to a national population health priority**: Federal policymakers must shift the regulatory perspective of diabetic limb preservation from a narrowly specialized clinical concern to a macroeconomic, population-level health metric to curtail billions in downstream public spending while meeting federal target goals.^8^

## Conclusion

Preventable diabetic limb loss represents a structural habit of reimbursing acute deterioration more predictably than early prevention.^4,5^ While clinical therapeutics have advanced, the healthcare system continues to absorb billions in avoidable spending due to a reactive delivery model.^1-3^ Correcting this costly blind spot requires moving toward a unified, prevention-first policy architecture. The empirical evidence confirms that early symptom recognition and coordinated preventive care can dramatically outperform federal benchmarks.^8^ The central challenge is no longer whether preventive diabetic limb preservation is clinically feasible but whether US health policy will systematically prioritize upstream preventive infrastructure before irreversible, high-cost harm occurs.

## Supplementary Material

qxag190_Supplementary_Data
